# Perfect Eyes

**Published:** 1900-12

**Authors:** 


PERFECT EYES. A Complete and Comprehensive Treatise on the Organ of Vision. Duodecimo, pp. 71. Illustrated. Sixth edition. New York: Benjamin F. Stephens, Publisher. 1900.

This peculiar work was probably intended for the laity. There are three pages of extracts from poems, containing some reference to the eye; following this the anatomy of the eye is briefly given, then follows a hodge-podge of cuts of astigmatic tests, curiosities of vision, mixed up with colored illustrations of the eye muscles, blood supply, and the like. Such an arrangement is entirely out of place and strikes one as absurd. The most advanced ophthalmologist will be pleased to learn that cataract is curable by massage, also strabismus, photophobia, amblyopia and choroiditis. All of which sounds like the advance sheets of an osteopathic manual. Beginning with the seventh chapter the book is interesting and well written, although too much stress is occasionally laid upon the value of massage. The author's warning against needless muscle-cutting and the optician are well expressed and worthy of praise. It is unfortunate with the mass of material that it has not been better arranged. The book is daintily gotten up,—the paper good and the cuts exceptionally good. There should be a steady demand for such a work and we hope to see a revised edition.—R. H. S.

